# Long-Term Organic–Inorganic Fertilization Regimes Alter Bacterial and Fungal Communities and Rice Yields in Paddy Soil

**DOI:** 10.3389/fmicb.2022.890712

**Published:** 2022-06-27

**Authors:** Tengfei Ma, Xiaohui He, Shanguo Chen, Yujia Li, Qiwei Huang, Chao Xue, Qirong Shen

**Affiliations:** ^1^Jiangsu Provincial Key Lab for Organic Solid Waste Utilization, Jiangsu Collaborative Innovation Center for Solid Organic Wastes, Educational Ministry Engineering Center of Resource-Saving Fertilizers, Nanjing Agricultural University, Nanjing, China; ^2^Key Laboratory of Green Intelligent Fertilizer Innovation MARD, Sinong Bio-organic Fertilizer Institute, Nanjing, China

**Keywords:** fertilization regimes, soil biological fertility, co-occurrence network, microbial community, rice

## Abstract

Microorganisms are the most abundant and diverse organisms in soils and have important effects on soil fertility. In this study, effects of the long-term fertilization treatments no fertilizer (CK), chemical fertilizer (nitrogen–phosphorus–potassium (NPK)), and organic–inorganic fertilizer (NPK and organic fertilizer (NPKM)) on rice yield and soil bacterial and fungal community diversity, structure, composition, and interaction networks were evaluated. Of the three treatments, the highest rice yield was in NPKM. Bacterial richness was significantly higher in NPKM than in NPK. Fertilization treatment significantly altered β diversity of communities, species composition of bacterial and fungal communities, and structure of soil microbial networks. The most complex bacterial and fungal interaction co-occurrence network with the highest average degree and numbers of edges and nodes was in NPKM. Relative abundance of the plant growth-promoting fungus *Trichoderma* increased significantly in NPKM compared with CK and NPK. The results of the study indicate that bacterial richness and microbial community member interactions (network complexity) might be suitable indicators of soil biological fertility. This research provides new insights on the effects of different fertilization regimes on responses of soil bacterial and fungal communities and their contributions to crop yield. New indicators such as bacterial richness and complexity of microbial interaction networks are also identified that can be used to evaluate soil biological fertility.

## Introduction

Chemical fertilizer is often used to compensate the loss of soil nutrients during growing stages of crops in which chemical fertility is the primary focus. By contrast, organic fertilizer is typically applied as a base fertilizer before crop growth. Many studies indicate that organic fertilizer plus chemical fertilizer treatment can increase yields compared with only chemical fertilization, even when total nitrogen (N), phosphorus (P), and potassium (K) contents are the same (Pan et al., 2009; Xie et al., 2016; Zhao et al., 2016). Addition of organic fertilizer with chemical fertilizer can slow the release of nutrients in the chemical fertilizer and thus reduce N loss and increase N use efficiency (Pan et al., 2009). The combination of fertilizer types can also optimize soil aggregate structure by increasing organic matter and thus improve soil physical structure (Bronick and Lal, 2005). However, how addition of organic fertilizers influences soil biological fertility has not been determined.

Microorganisms are the most abundant and diverse soil organisms. On average, 1 g soil contains 10^10^ cultivable cells and approximately 10^4^ microbial species (Liu et al., 2012; Saleem et al., 2019). As the most active component of an ecosystem, microorganisms have essential roles in energy flow, particularly in decomposition of organic matter, and biogeochemical cycling of nutrient elements (Morris and Blackwood, 2015). Therefore, growth and metabolism of microbial communities can directly and indirectly affect crop growth (Ahn et al., 2012). The most significant effects of soil microorganisms are on the decomposition of organic matter and the release of minerals. Soil microorganisms regulate decomposition of organic matter and release of minerals and are essential in forming humus and improving structure and cultivability of soil, which ultimately promote crop growth. Simultaneously, crops secrete biosynthesized organic matter and other substances into soils through the root system to provide soil microorganisms with sources of nutrients such as carbon (C) and N. Those root exudates can enrich and increase the growth of bacterial flora that are beneficial to plant growth and help resist infection by pathogenic bacteria (Hou et al., 2021). Thus, soil microbial activities and crop growth and yield are interdependent, and it is therefore important to consider activities of microorganisms when conducting research on crop yields in agroecosystems.

In agroecosystems, soil microbial biomass and diversity are potential indicators of soil quality (Bending et al., 2004), which is associated with soil productivity and crop growth and yield and can reflect soil vitality, health status, and biological fertility. Soil biological fertility is the core component of soil fertility, the key to sustainable use of agricultural land, and the foundation of a future agricultural revolution (Hatfield and Walthall, 2015). As decomposers and material cyclers of soil ecosystems, soil microorganisms are the core of soil biological fertility and are essential in regulating biological fertility. However, microbial community indicators that can be used to evaluate soil biological fertility have not been determined. To date, most studies have focused on effects of different fertilization treatments on soil bacterial communities (Gu et al., 2009; Zhao et al., 2014, 2016), whereas effects on soil fungal communities and their functions have largely been ignored. Many studies have examined how different fertilization regimes shape soil microbial community diversity, structure, and composition (Ge et al., 2008; Gu et al., 2009; van der Bom et al., 2018). However, few studies focus on interactions among community members. Community member interactions are largely dependent on nutrient and energy supplies in a soil (Qiu et al., 2021) and therefore might be an important indicator of soil biological fertility.

In this study, a long-term field fertilization experiment was set up in paddy field that included three treatments: no fertilization, chemical fertilization, and chemical fertilization plus organic fertilizer addition. In addition to determining rice yields, high-throughput sequencing was used to investigate diversity, structure, composition, and interaction networks of soil bacterial and fungal communities. The aims of the study were to understand how different fertilization regimes influence soil microbial communities and rice yields and to determine which microbial community parameters might be used as indicators of soil biological fertility.

## Materials and Methods

### Site Description

The study was conducted in a long-term experimental field site in Changshu, Jiangsu Province, China (31°18′N, 120°37′E). The site is at the center of the Tai Lake plain region, where the cropping regime is a rotation of summer paddy rice and winter wheat. The climate is humid subtropical monsoon with average annual rainfall of 1,063 mm and annual mean minimum and maximum temperatures of 3.1°C and 33°C, respectively (Chen et al., 2016; Wang et al., 2016a). The field was tilled to an average depth of 20 cm before either sowing wheat or transplanting rice seedlings. Rice plots were flooded with 5 cm of standing water from July to September (Wang et al., 2016b). Rice was transplanted in June using two seedlings per hill at 13 cm × 28 cm spacing (Wang et al., 2016a). Following rice harvest, wheat was sown with seeds at 150 kg ha^−1^ in October or November every year. A sickle was used to manually harvest crops at ground level, and aboveground biomass was removed from plots. Fertilizers were applied as basal fertilizer after harvest of both rice and wheat (Wang et al., 2016b).

### Field Experiment and Soil Sampling

Three treatments with four replicates were established in a randomized block design in 2005, and each replicate plot was 16 m^2^ (4 × 4 m). To avoid margin effects, yield was measured in a 4-m^2^ (2 m × 2 m) area only in the middle of each plot, with yield converted to yield per hectare. Experimental treatments included CK (no fertilizer control), NPK (240 kg N ha^−1^, 90 kg P_2_O_5_ ha^−1^, and 120 kg K_2_O ha^−1^), and NPKM (The NPK and NPKM treatments contained the same total amounts of nutrients. NPKM contained 4,500 kg ha^−1^ organic fertilizer). The organic fertilizer was derived from composted pig manure with rice straw and contained 26.4% organic C, 2.5% total N, 1.6% P (P_2_O_5_), and 1.3% K (K_2_O).

Soil samples were collected at 0 to 20 cm after rice harvest in October 2015. Four soil cores (5-cm diameter) were randomly collected in each 4 × 4-m plot. Soil samples from different treatments were mixed separately and sieved (2 mm) to remove plant materials, roots, and stones. To minimize changes in soil communities after sampling, DNA was immediately extracted from fresh soil. Subsamples were frozen at −80°C.

### DNA Extraction

Soil DNA was extracted from 0.25 g of fresh soil with a MoBio Powersoil^™^ DNA Isolation Kit (MoBio Laboratories, Carlsbad, CA, United States of America) using the bead-based homogenizer protocol according to the manufacturer’s instructions. Quantity and quality of DNA extracts were assayed by a Nanodrop ND-2000 UV–VIS Spectrophotometer (NanoDrop Technologies, Wilmington, DE, United States of America), and DNA was stored at −80°C until analysis.

### Gene Amplification and Deep Sequencing

Amplification of the 16S rRNA gene V4 hypervariable region was performed using a PCR reaction solution containing 12.5 μl of Master Mix (Qiagen Inc., Valencia, CA, United States of America), 0.5 μl (10 mM) of 515F primer (5′-GTGCCAGCMGCCGCGGTAA-3′), 0.5 μl (10 mM) of 806R primer (5′-GGACTACHVGGGTWTCTAAT-3′; Caporaso et al., 2012), 1 μl of DNA template (10 ng μl^−1^), and 10.5 μl of ddH_2_O to a final volume of 25 μl. The PCR protocol was performed in triplicate using the following conditions: 10 min at 95°C for initial denaturing, followed by 35 cycles of 95°C for 15 s, 56°C for 15 s, and 72°C for 30 s, with a final extension at 72°C for 5 min.

Amplification of the fungal ITS1 (Internal Transcribed Spacer I) region was performed using a PCR reaction solution containing 12.5 μl of Master Mix (Qiagen Inc.), 0.5 μl (10 mM) of ITS1F primer (5′-CTTGGTCATTTAGAGGAAGTAA-3′; Gardes and Bruns, 1993), 0.5 μl (10 mM) of ITS2 primer (5′-GCTGCGTTCTTCATC-GATGC-3′; Baldwin, 1992), 1 μl of DNA template (10 ng μl^−1^), and 10.5 μl of ddH_2_O to a final volume of 25 μl. The PCR protocol was performed in triplicate using the following conditions: 10 min at 95°C for initial denaturing, followed by 35 cycles of 95°C for 15 s, 56°C for 15 s, and 72°C for 30 s, with a final extension at 72°C for 5 min. The Illumina sequencing adapter-ligated reverse primer contained a 6-bp bar code specific to each sample for identification (Caporaso et al., 2012). After amplification, triplicate PCR products were pooled and purified using a PCR Cleanup Kit (Axygen Biosciences, Union City, CA, United States of America). Bacterial and fungal PCR products were pooled separately for sequencing. Sequencing was performed on a single lane of an Illumina MiSeq platform at Personal Biotechnology Co., Ltd. (Shanghai, China). All sequence data have been deposited in the NCBI (National Center for Biotechnology Information) Sequence Read Archive database. The accession numbers are SRP359136 for bacterial data and SRP359138 for fungal data.

### Bioinformatics Analysis and Statistics of High-Throughput Sequencing Data

High-throughput sequencing data were processed using USEARCH v.10.0 (Edgar, 2010), VSEARCH v.2.13 (Rognes et al., 2016), and in-house scripts (Zhang et al., 2018). First, the --fastq_mergepairs command was used to merge paired-end sequences of the sequencing data and rename them. Then, the --fastx_filter command was used to remove the double-ended primers and bar codes and to perform quality control to make the error rate less than 1%. The --derep_fulllength command was used to reduce sequence redundancy. Redundant sequences were clustered into operational taxonomic units (OTU) with 97% similarity by using the -cluster_otus command, and chimeras were removed simultaneously. UPASE (Edgar, 2013) was used to select representative sequences, and then an OTU table was generated by the --usearch_global command. Species annotations were conducted using the -sintax command of VSEARCH with the SILVA database (Quast et al., 2013) for bacteria and the UNITE database (Abarenkov et al., 2010) for fungi.

Bacterial and fungal sequences were flattened to 3,935 and 5,837 sequences per sample, respectively, before α diversity indices were calculated. Chao1, Pielou’s evenness, and Shannon indices were calculated to evaluate community richness, evenness, and diversity (Lu et al., 2020), respectively. Principal coordinate analysis (PCoA; Zhang et al., 2019) was used to show differences in microbial community structure among samples. To test the significance of differences in microbial community structure among different treatments, adonis analysis based on permutational multivariate analysis of variance (PERMANOVA; Zhang et al., 2019) was used. One-way ANOVA followed by Tukey’s *post hoc* test was performed to compare means of rice yield, α-diversity indices, microbial abundance, and the Proteobacteria to Acidobacteria ratio. Ecological networks of fungi and bacteria were constructed, and network topology parameters were calculated in R (https://www.r-project.org/) using the “igraph” package (Wang et al., 2018). Pearson correlation analyses were performed to examine relations between rice yield and α diversity indices. Mantel tests (Wang et al., 2016b) were conducted to test correlations between rice yield and community composition. All data visualization was performed in R using the “ggplot2” package. When considering bacteria and fungi together, OTU tables of fungi and bacteria were combined for calculations.

## Results

### Effects of Different Long-Term Fertilization Treatments on Rice Yield

**Figure 1:**
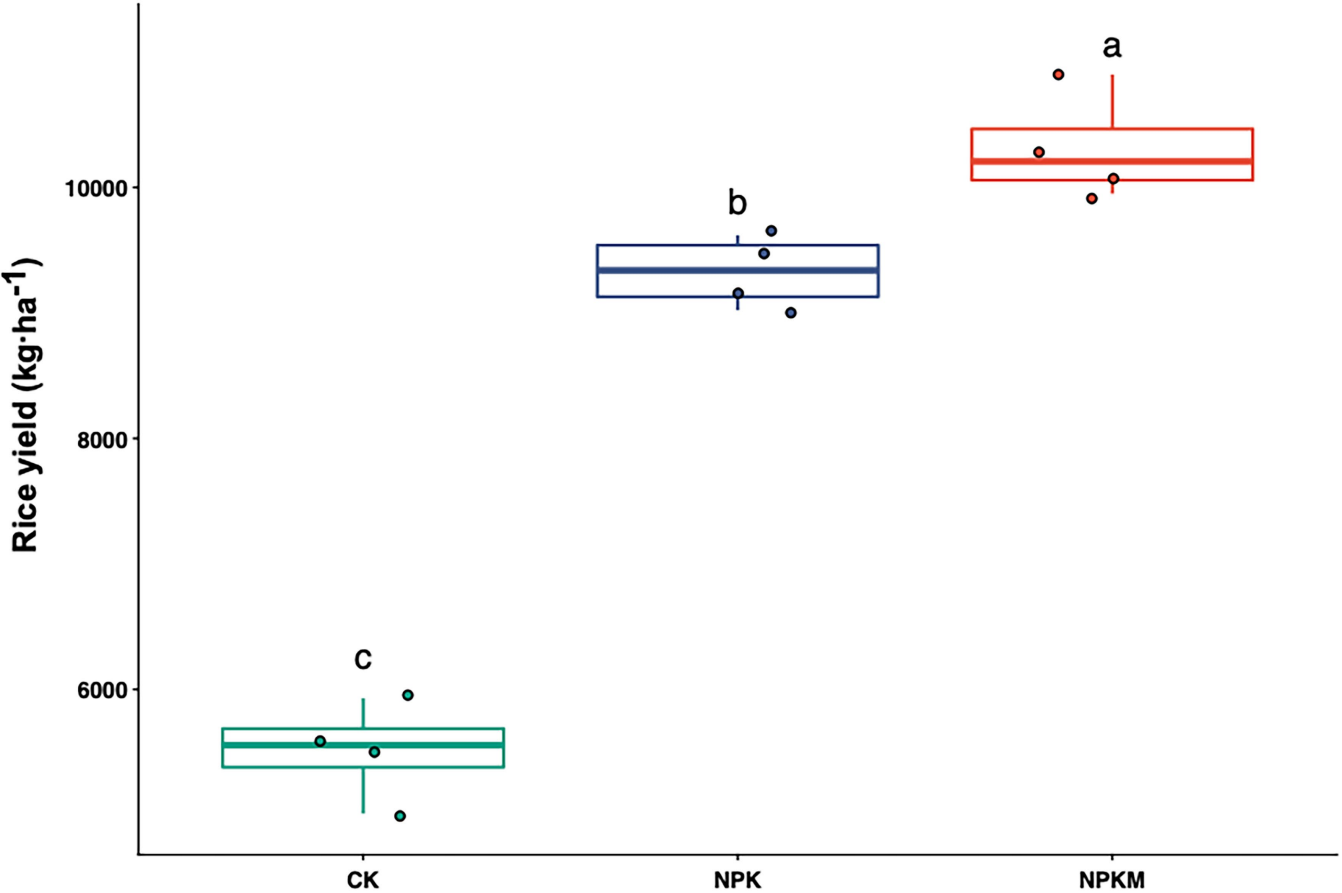
Effects of long-term fertilization treatments on rice yield. Different letters indicate significant differences among treatments (Tukey’s *post hoc* test, *p* < 0.05).

Rice yield varied among different fertilization treatments (Figure 1). Compared with CK, fertilization treatments significantly increased rice yield (Tukey’s *post hoc* test, *p* < 0.05). Average rice yields were 5,513 kg/ha in CK, 9,329 kg/ha in NPK, and 10,316 kg/ha in NPKM. Although NKP and NPKM had equal inputs of N, P, and K, rice yield was significantly higher in NPKM than in NPK (Tukey’s *post hoc* test, *p* < 0.05).

### Effects of Different Fertilization Treatments on Microbial Alpha Diversity

**Figure 2:**
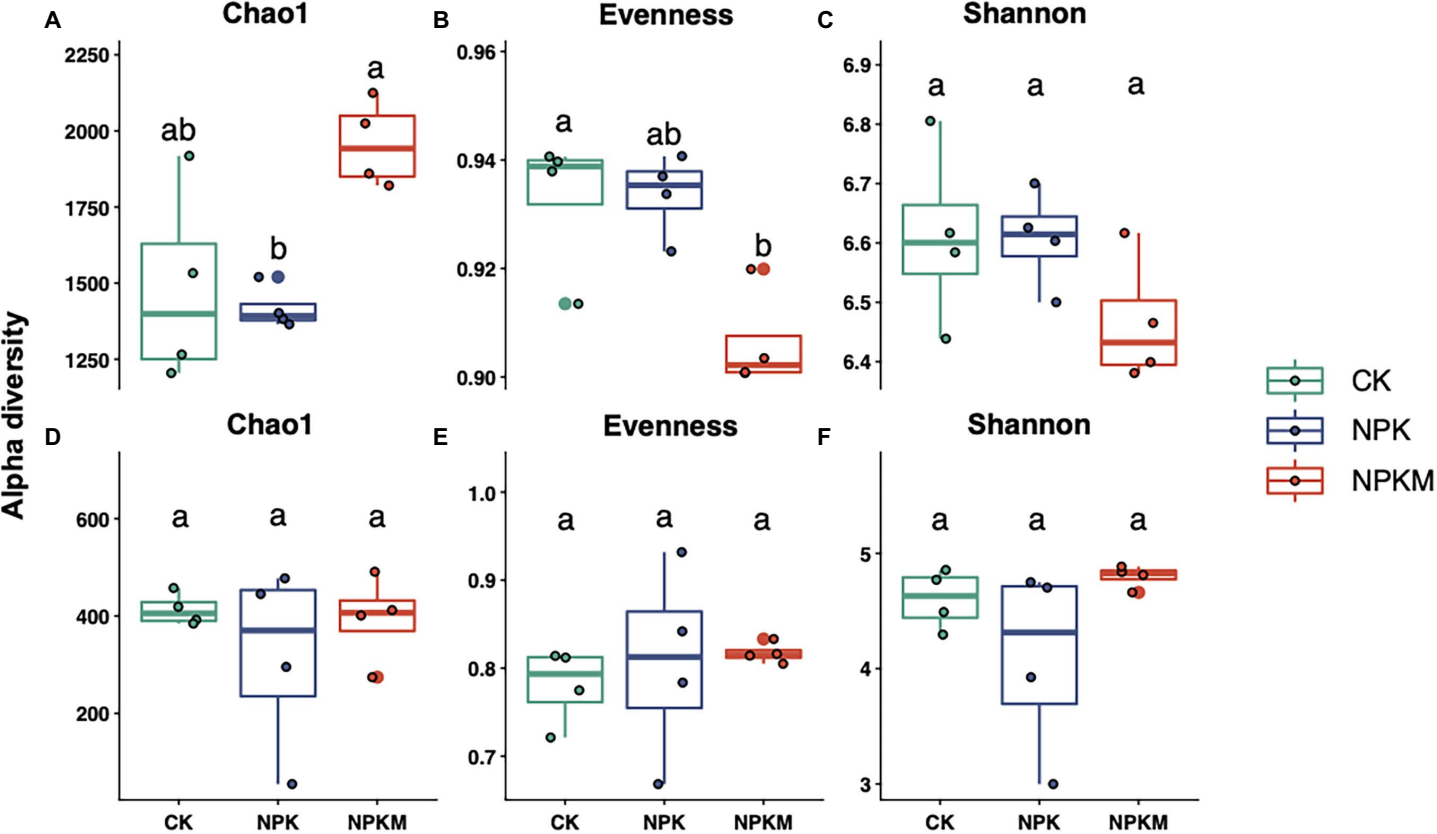
Effects of long-term fertilization treatments on Chao1 richness, evenness, and Shannon diversity indices of **(A–C)** bacteria and **(D–F)** fungi. Different letters indicate significant differences among treatments (Tukey’s *post hoc* test, *p* < 0.05).

Chao1, evenness, and Shannon indices were calculated to estimate microbial richness, evenness, and diversity, respectively (Figure 2). Compared with NPK, NPKM significantly increased bacterial richness (Tukey’s *post-hoc* test, *p* < 0.05) but decreased bacterial evenness and diversity, although differences were not significant. However, different fertilization treatments did not significantly affect fungal richness, evenness, and diversity. In Pearson correlation analyses, rice yield was positively correlated with bacterial richness and fungal evenness but negatively correlated with bacterial evenness and diversity and fungal richness and diversity, although none of the correlations were significant (Supplementary Figure S1).

### Effects of Different Fertilization Treatments on Microbial Beta Diversity

**Figure 3:**
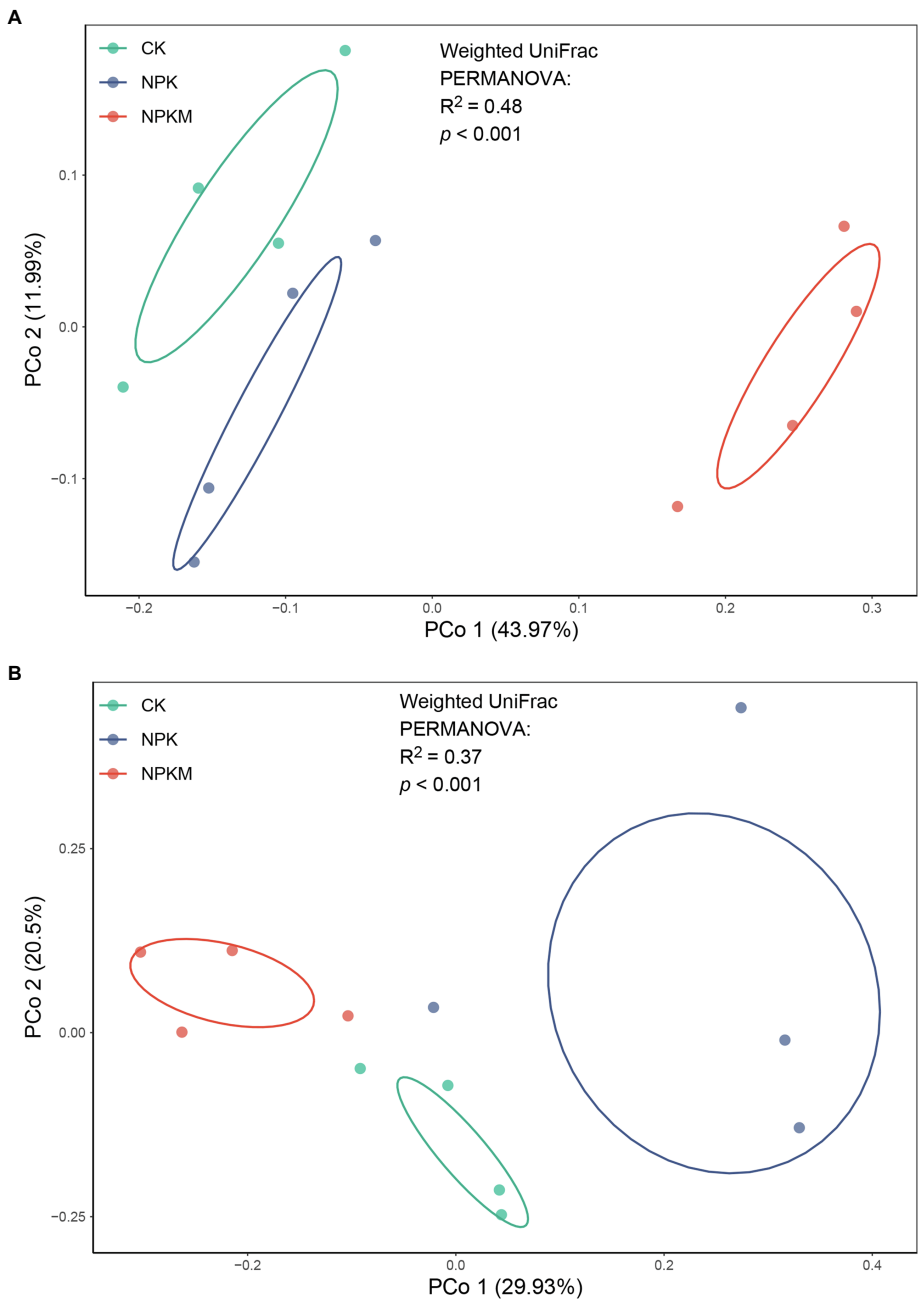
Unconstrained principal coordinate analysis (PCoA) with weighted UniFrac distance showing effects of long-term fertilization treatments on beta-diversity of soil **(A)** bacterial and **(B)** fungal communities.

In the unconstrained PCoA of weighted UniFrac distance, soil bacterial and fungal communities formed three distinct clusters according to different fertilization treatments (PERMANOVA: *p* < 0.01; Figure 3). The NPKM community was significantly different from those in CK and NPK and separated along the first coordinate axis, which indicated that the greatest variation among treatments was most likely due to the addition of organic fertilizer. According to Mantel tests, rice yield was significantly positively correlated with bacterial community structure (*r* = 0.462, *p* = 0.005), whereas fungal community structure and rice yield were not significantly correlated (*r* = 0.033, *p* = 0.408).

### Effects of Different Fertilization Treatments on Composition of Bacterial and Fungal Communities

**Figure 4:**
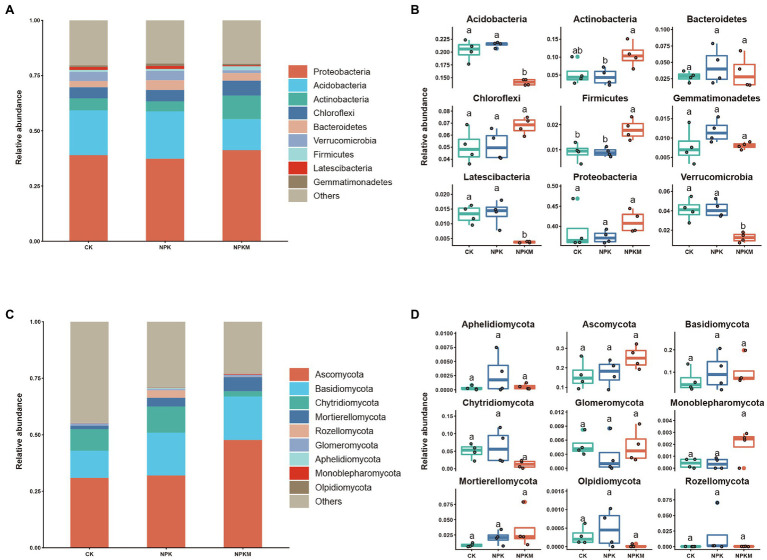
Effects of long-term fertilization treatments on composition of bacterial and fungal communities. Phylum-level composition of **(A)** bacterial and **(C)** fungal communities. Analysis of differences in relative abundances of **(B)** bacterial phyla in **A** and **(D)** fungal phyla in **C**. Names of the nine most abundant phyla are shown, and other phyla were grouped as “Others.” Different letters indicate significant differences (Tukey’s *post hoc* test, *p* < 0.05).

Fertilization treatments shaped bacterial community composition (Figure 4A). Among all samples, the nine most abundant phyla of bacteria were Proteobacteria (39.5%), Acidobacteria (19.1%), Actinobacteria (6.5%), Chloroflexi (5.4%), Bacteroidetes (3.3%), Verrucomicrobia (3.2%), Firmicutes (1.1%), Latescibacteria (1.1%), and Gemmatimonadetes (1.0%). Addition of organic fertilizer significantly decreased relative abundances of Acidobacteria, Verrucomicrobia, and Latescibacteria (Tukey’s *post hoc* test, *p* < 0.05) but significantly increased those of Actinobacteria and Firmicutes (Tukey’s *post hoc* test, *p* < 0.05; Figure 4B).

Fungal community composition was also altered by fertilization treatments (Figure 4C). Among all samples, the nine most abundant phyla of fungi were Ascomycota (20.2%), Basidiomycota (9.2%), Chytridiomycota (4.1%), Mortierellomycota (2.2%), Rozellomycota (0.7%), Glomeromycota (0.4%), Aphelidiomycota (0.1%), Monoblepharomycota (0.1%), and Olpidiomycota (0.03%).

**Figure 5:**
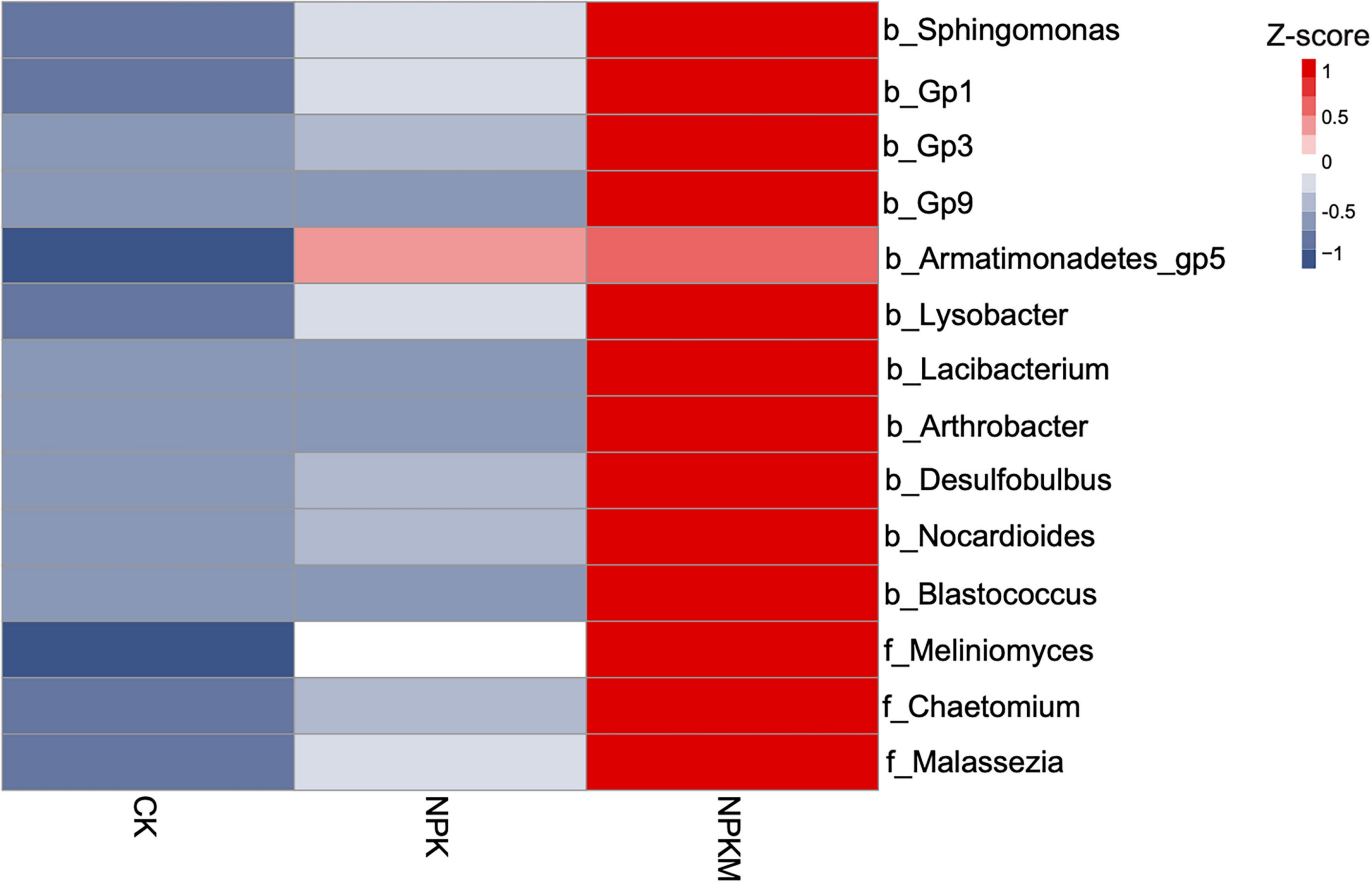
Heat map of relative abundances of genera of bacteria and fungi with positive correlations to rice yield. Relative abundances were *Z*-score normalized. Genus-level bacteria names begin with “b”, and genus-level fungi names begin with “f”.

Correlation analysis revealed a genus-level core microbial community that was significantly correlated with rice yield (Pearson, *p* < 0.05). The genera of bacteria were *Sphingomonas*, *Acidobacteria_Gp1*, *Acidobacteria_Gp3*, *Acidobacteria_Gp9*, *Armatimonadetes_Gp5*, *Lysobacter*, *Lacibacterium*, *Arthrobacter*, *Desulfobulbus*, *Nocardioides*, and *Blastococcus*, and the genera of fungi were *Meliniomyces*, *Chaetomium*, and *Malassezia* (Figure 5). Compared with CK and NPK, NPKM significantly increased relative abundances of *Tausonia* and *Trichoderma* (Tukey’s *post hoc* test, *p* < 0.05).

### Effects of Different Fertilization Treatments on Co-occurrence Networks of Soil Microorganisms

**Figure 6:**
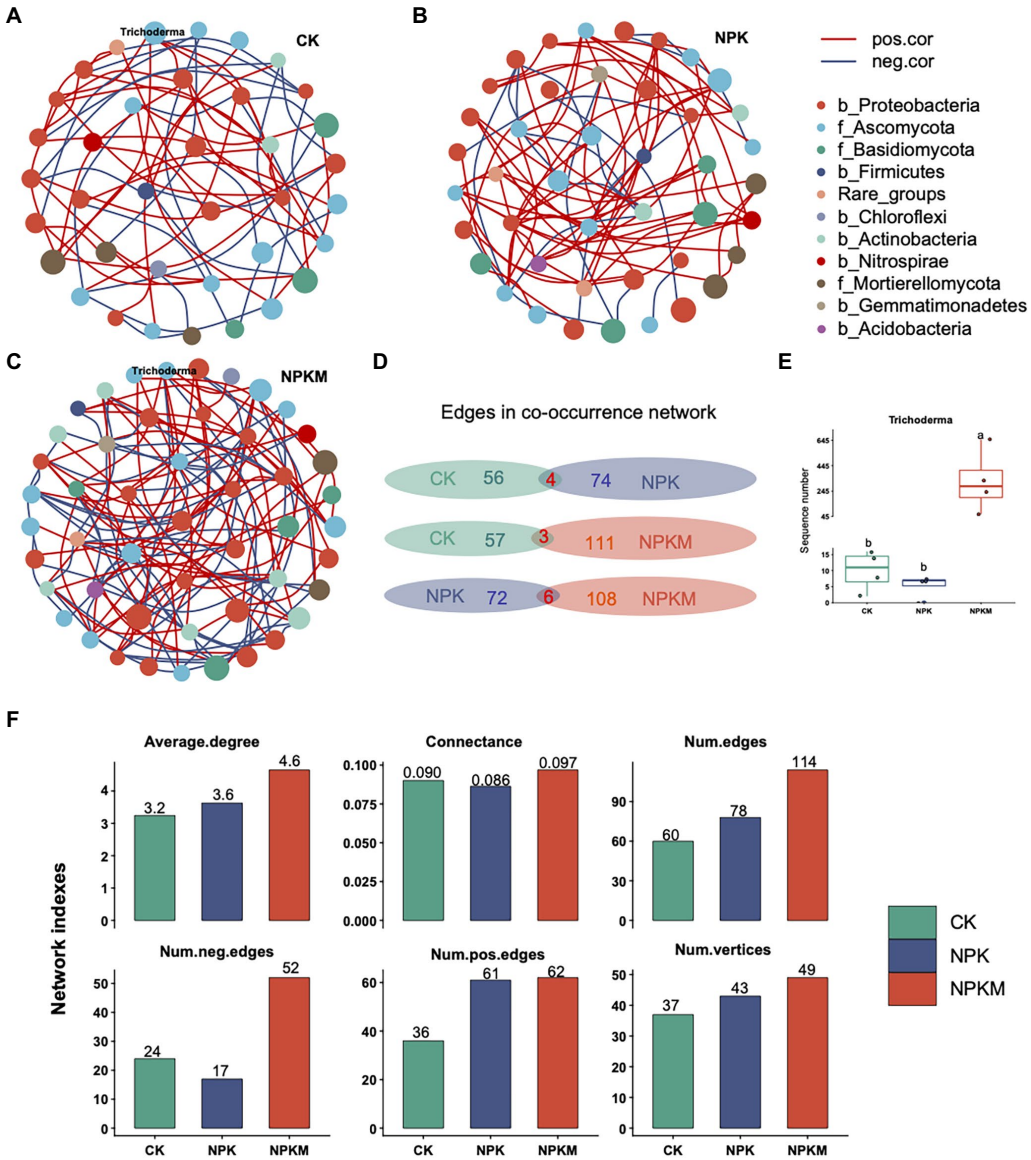
Co-occurrence networks of bacterial and fungal communities in **(A)** control (CK), **(B)** nitrogen–phosphorus–potassium (NPK), and **(C)**, NPK plus organic fertilizer (NPKM) fertilization treatments. Each node in a network represents a genus of bacteria or fungi. Size of a node represents average relative abundance of a genus of bacteria or fungi. Different colors of nodes provide taxonomic information at the phylum-level. In the legend, names of bacterial phyla begin with “b,” and names of fungal phyla begin with “f.” **(D)** Common and unique edges in co-occurrence networks between each two of the three treatments. **(E)** Comparison of sequence numbers of the fungus *Trichoderma* in different fertilization treatments. **(F)** Indices related to topological structure of the co-occurrence interaction network of bacterial–fungal communities.

Nodes in the bacterial and fungal co-occurrence network included seven bacterial phyla (Proteobacteria, Actinobacteria, Chloroflexi, Firmicutes, Nitrospirae, Gemmatimonadetes, and Acidobacteria) and three fungal phyla (Ascomycota, Basidiomycota, and Mortierellomycota; Figure 6). A unique microbial co-occurrence network formed in each treatment. The most complex network was in NPKM, followed by those in NPK and CK. Compared with CK and NPK, the NPKM network had the highest connectivity (0.097), number of edges (114), number of vertices (49), and average degree (4.6; Figure 6).

To quantify differences in co-occurrence networks among treatments, numbers of common and unique edges of CK, NPK, and NPKM networks were calculated. There were few common edges shared by any two treatments, compared with many unique edges in each treatment. Networks in CK and NPK shared only four edges, whereas the CK network had 56 unique edges and the NPK network had 74. Networks in CK and NPKM shared only three edges, whereas the CK network had 57 unique edges and the NPKM network had 111. Networks in NPK and NPKM shared only six edges, but the NPK network had 72 unique edges and the NPKM network had 108 (Figure 6).

The fungal genus *Trichoderma* was absent in NPK but occurred in NPKM. The sequence number of *Trichoderma* was significantly higher in NPKM than in CK and NPK (Tukey’s *post hoc* test, *p* < 0.05), with the lowest number in NPK (Figure 6).

## Discussion

Soil microbial biomass and diversity are potential indicators of soil quality (Bending et al., 2004), as well as important factors in maintaining integrity and stability of soil functions in agroecosystems (Kennedy and Smith, 1995; Nannipieri et al., 2003; Brussaard et al., 2007). They are associated with the level of soil biological fertility and thereby affect crop yields. In this study, in a long-term field fertilization experiment, effects of no fertilization, chemical fertilization, and chemical fertilization plus organic fertilizer treatments on rice yields and soil bacterial and fungal communities were evaluated. High-throughput sequencing was used to evaluate composition of microbial communities. The highest rice yield among the three treatments was in NPKM. Compared with NPK, combined application of organic and inorganic fertilizers significantly increased bacterial richness but decreased bacterial evenness. Those results suggested that application of organic fertilizer increased the number of species to increase bacterial richness and enriched certain groups that preferred organic fertilizer to decrease bacterial evenness. By contrast, fertilization treatments did not significantly affect indices of fungal alpha diversity. Thus, fertilizer treatments had different effects on alpha diversity of bacterial and fungal communities. The results are consistent with those of previous studies in which organic fertilizer additions increased soil microbial abundance but not evenness (Parham et al., 2003; Sun et al., 2004; Jangid et al., 2008; Yuan et al., 2008).

Fertilization can stimulate growth of soil microorganisms and thereby affect microbial community structure (Chu et al., 2007; Esperschütz et al., 2007; Gu et al., 2009). In previous studies, long-term application of different fertilizers significantly altered the structure of soil bacterial and fungal communities (Allison et al., 2007; Wu et al., 2011; Yu et al., 2013). According to the PCoA in this study, the different fertilization treatments altered bacterial and fungal species composition and therefore microbial community structure. Mantel tests indicated that rice yield was positively correlated with bacterial community structure but not with fungal community structure. Therefore, because the response of bacterial communities to fertilization treatments was greater than that of fungal communities, bacterial community parameters might be suitable indicators of soil biological fertility.

**Figure 7:**
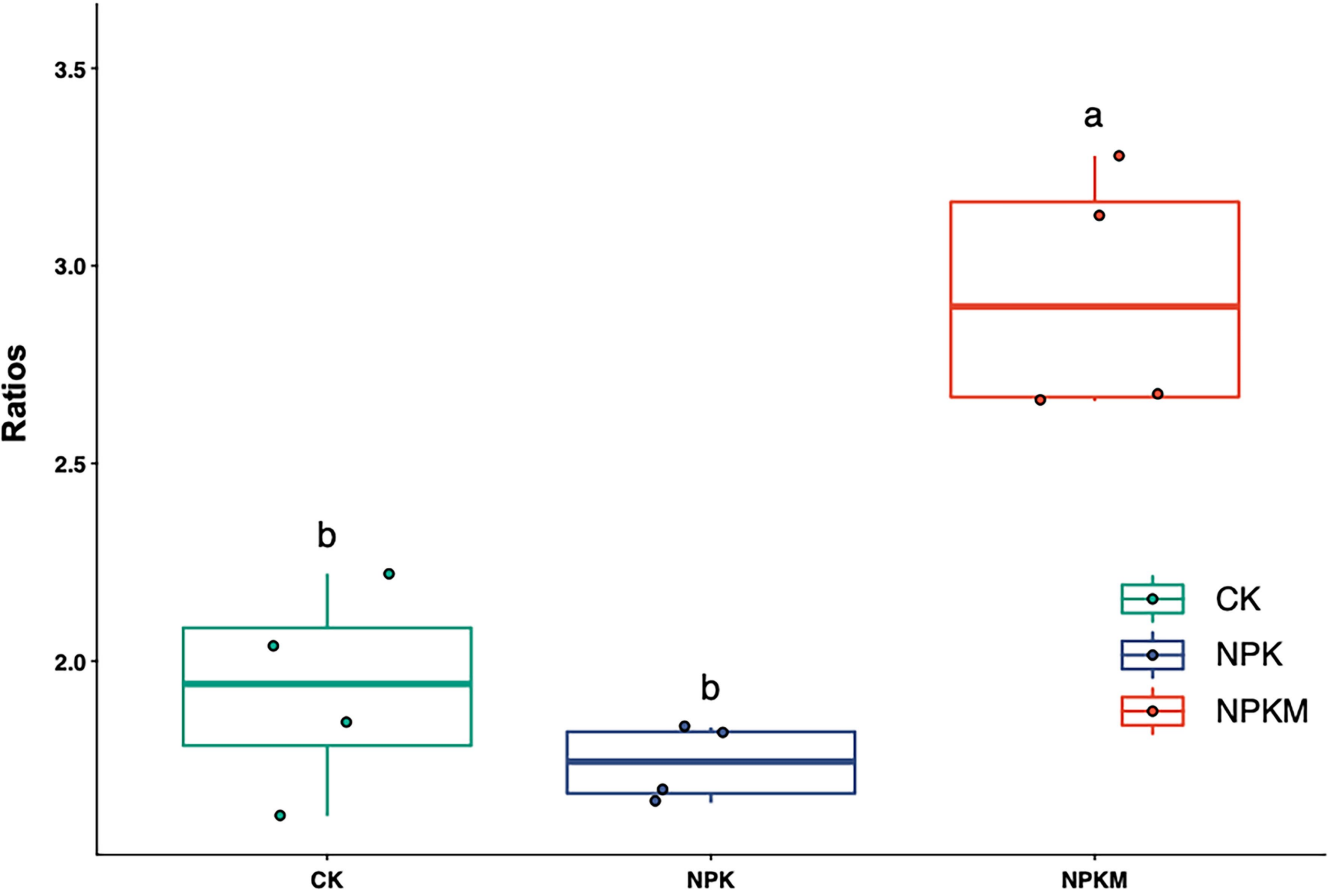
Effects of long-term fertilization treatments on the ratio of *Proteobacteria* to *Acidobacteria*. Different letters indicate significant differences (Tukey’s *post hoc* test, *p* < 0.05).

Bacteria can be divided into two life types according to life history strategy, namely copiotroph *r*-strategists and oligotroph *K-*strategists (Pianka, 1970; Fierer et al., 2007). When soil organic matter content is high, *r*-strategists are usually the primary decomposers of organic matter, and microorganisms in the eutrophic group are most abundant. By contrast, when soil organic matter content is low and nutrients are lacking, *K*-strategists in the oligotrophic group have a competitive advantage (Fontaine et al., 2003). Actinobacteria, α-Proteobacteria, β-Proteobacteria, and Bacteroidetes are generally regarded as *r-*strategists in the eutrophic group and are generally more abundant in high-fertility soils (Fierer et al., 2007; Newton and Mcmahon, 2011; Leff et al., 2015; Zhou et al., 2015). Acidobacteria and Verrucomicrobia are classified as K-strategists in the oligotrophic group (Ramirez et al., 2010). In a previous study, application of organic fertilizer significantly increased relative abundances of r-strategists in the eutrophic group, such as Proteobacteria, Bacteroidetes, and Actinobacteria, whereas application of inorganic fertilizers significantly increased relative abundances of K-strategists in the oligotrophic group, such as Acidobacteria (Xun et al., 2016). In this study, the three most abundant bacterial groups were Proteobacteria (39.5%), Acidobacteria (19.1%), and Actinobacteria (6.5%), which together accounted for 65.1% of the bacterial community. In NPKM, relative abundances of *r*-strategists in the eutrophic group (Actinobacteria and Proteobacteria) increased, but those of K-strategists in the oligotrophic group (Acidobacteria and Verrucomicrobia) decreased significantly (Figure 4). The ratio of abundances of Proteobacteria to Acidobacteria may be an indicator of nutrient status of a soil ecosystem (Smit et al., 2001; Torsvik and Øvreås, 2002). In this study, the ratio of Proteobacteria to Acidobacteria was significantly higher in NPKM than in other treatments (Figure 7), indicating that soil nutrient status and productivity were also high. However, it should be noted that considering microbial community response at finer resolutions (e.g., family, genus and species level) may be more adequate when assigning life strategies to microorganisms (Ho et al., 2017). Notably, abundance of the fungal genus *Trichoderma* increased significantly in NPKM (Tukey’s *post hoc* test, *p* < 0.05; Figure 6E). *Trichoderma* is a well-known plant growth-promoting fungus (Masunaka et al., 2011) that can significantly increase crop growth and productivity (Hyakumachi, 1994). Thus, *Trichoderma* might be one reason for the high rice yield in the organic–inorganic combined treatment.

Co-occurrence network analysis was conducted to examine interactions among soil microorganisms in response to different fertilization treatments. The most complicated network was in NPKM, suggesting organic fertilizer addition increased interactions among microbial community members (Figure 6). The increase in complexity was likely due to the additional C input in the organic fertilizer. Soil bacteria and fungi can use those carbon sources to generate additional energy and thus boost expression of soil microbial functions, which increases the number of microbial interactions. The results suggested that organic fertilizer addition increased stability of community structure and therefore ability to resist external interference. In addition, by increasing levels of cooperation within microbial communities, addition of organic fertilizer was likely conducive to developing soil biological fertility. Thus, complexity of microbial interaction networks might be an indicator of soil biological fertility.

Notably, the fungal genus *Trichoderma* was not in the co-occurrence network in NPK but was in that in NPKM. Enrichment of the beneficial fungus *Trichoderma* in the combination organic and inorganic treatment indicated there were changes in the interaction network of soil microorganisms that could increase rice productivity. Inoculation of *Trichoderma* could be used to improve crop productivity in agroecosystems.

## Conclusion

Soil biological fertility is an important component of overall soil fertility. However, how to best evaluate soil biological fertility has not been determined. Soil microbial communities are important in regulating soil biological fertility. Thus, the responses of soil bacterial and fungal community diversity, structure, composition, and interactions to different fertilization treatments were analyzed by high-throughput sequencing. The inorganic and organic fertilizer treatment had the highest bacterial richness, the most unique bacterial and fungal communities due to species selection by fertilizers, and the microbial network with the highest complexity, and as a result, the highest productivity. *Trichoderma* was enriched in the NPKM treatment and might be a key contributor to the increase in soil fertility. The results indicate that bacterial richness and complexity of microbial interaction networks could be used as indicators of soil biological fertility. This research provides new insights on responses of soil bacterial and fungal communities to different fertilization treatments and their contributions to crop yield. The study also identifies new indicators to evaluate soil biological fertility and indicates that inoculation with *Trichoderma* might improve crop productivity in agroecosystems.

## Data Availability Statement

The datasets presented in this study can be found in online repositories. The names of the repository/repositories and accession number(s) can be found in the article/Supplementary Material.

## Author Contributions

CX, QH, and QS designed this experiment. TM, XH, SC, and YL carried out the experiment. TM wrote this manuscript. CX and QS revised the manuscript. All authors contributed to the article and approved the submitted version.

## Funding

This work was funded by the China Postdoctoral Science Foundation (2017M621761 and 2018T110510).

## Conflict of Interest

The authors declare that the research was conducted in the absence of any commercial or financial relationships that could be construed as a potential conflict of interest.

## Publisher’s Note

All claims expressed in this article are solely those of the authors and do not necessarily represent those of their affiliated organizations, or those of the publisher, the editors and the reviewers. Any product that may be evaluated in this article, or claim that may be made by its manufacturer, is not guaranteed or endorsed by the publisher.

## References

[ref1] Abarenkov K. Nilsson R. H. Larsson K. H. Alexander I. J. Eberhardt U. Erland S.. (2010). The UNITE database for molecular identification of fungi – recent updates and future perspectives. New Phytol. 186, 281–285. doi: 10.1111/j.1469-8137.2009.03160.x, PMID: 20409185

[ref2] Ahn J. H. Song J. Kim B. Y. Kim M. S. Joa J. H. Weon H. Y. (2012). Characterization of the bacterial and archaeal communities in rice field soils subjected to long-term fertilization practices. J. Microbiol. 50, 754–765. doi: 10.1007/s12275-012-2409-6, PMID: 23124742

[ref3] Allison S. D. Hanson C. A. Treseder K. K. (2007). Nitrogen fertilization reduces diversity and alters community structure of active fungi in boreal ecosystems. Soil Biol. Biochem. 39, 1878–1887. doi: 10.1016/j.soilbio.2007.02.001

[ref4] Baldwin B. G. (1992). Phylogenetic utility of the internal transcribed spacers of nuclear ribosomal DNA in plants: an example from the Compositae. Mol. Phylogenet. Evol. 1, 3–16. doi: 10.1016/1055-7903(92)90030-K, PMID: 1342921

[ref5] Bending G. D. Turner M. K. Rayns F. Marx M.-C. Wood M. (2004). Microbial and biochemical soil quality indicators and their potential for differentiating areas under contrasting agricultural management regimes. Soil Biol. Biochem. 36, 1785–1792. doi: 10.1016/j.soilbio.2004.04.035

[ref6] Bronick C. J. Lal R. (2005). Soil structure and management: a review. Geoderma 124, 3–22. doi: 10.1016/j.geoderma.2004.03.005

[ref7] Brussaard L. Ruiter P. Brown G. G. (2007). Soil biodiversity for agricultural sustainability. Agric. Ecosyst. Environ. 121, 233–244. doi: 10.1016/j.agee.2006.12.013

[ref8] Caporaso J. G. Lauber C. L. Walters W. A. Berg-Lyons D. Huntley J. Fierer N.. (2012). Ultra-high-throughput microbial community analysis on the Illumina HiSeq and MiSeq platforms. ISME J. 6, 1621–1624. doi: 10.1038/ismej.2012.8, PMID: 22402401 PMC3400413

[ref9] Chen C. Zhang J. Lu M. Qin C. Chen Y. Yang L.. (2016). Microbial communities of an arable soil treated for 8 years with organic and inorganic fertilizers. Biol. Fertil. Soils 52, 455–467. doi: 10.1007/s00374-016-1089-5

[ref10] Chu H. Lin X. Fujii T. Morimoto S. Yagi K. Hu J.. (2007). Soil microbial biomass, dehydrogenase activity, bacterial community structure in response to long-term fertilizer management. Soil Biol. Biochem. 39, 2971–2976. doi: 10.1016/j.soilbio.2007.05.031

[ref11] Edgar R. C. (2010). Search and clustering orders of magnitude faster than BLAST. Bioinformatics 26, 2460–2461. doi: 10.1093/bioinformatics/btq461, PMID: 20709691

[ref12] Edgar R. C. (2013). UPARSE: highly accurate OTU sequences from microbial amplicon reads. Nat. Methods 10, 996–998. doi: 10.1038/nmeth.2604, PMID: 23955772

[ref13] Esperschütz J. Gattinger A. Mder P. Schloter M. Fließbach A. (2007). Response of soil microbial biomass and community structures to conventional and organic farming systems under identical crop rotations. FEMS Microbiol. Ecol. 61, 26–37. doi: 10.1111/j.1574-6941.2007.00318.x, PMID: 17442013

[ref14] Fierer N. Bradford M. A. Jackson R. B. (2007). Toward an ecological classification of soil bacteria. Ecology 88, 1354–1364. doi: 10.1890/05-1839 17601128

[ref15] Fontaine S. Mariotti A. Abbadie L. (2003). The priming effect of organic matter: a question of microbial competition? Soil biol. Biochemist 35, 837–843. doi: 10.1016/S0038-0717(03)00123-8

[ref16] Gardes M. Bruns T. D. (1993). ITS primers with enhanced specificity for basidiomycetes - application to the identification of mycorrhizae and rusts. Mol. Ecol. 2, 113–118. doi: 10.1111/j.1365-294X.1993.tb00005.x, PMID: 8180733

[ref17] Ge Y. Zhang J.-b. Zhang L.-m. Yang M. He J.-z. (2008). Long-term fertilization regimes affect bacterial community structure and diversity of an agricultural soil in northern China. J. Soils Sediments 8, 43–50. doi: 10.1065/jss2008.01.270

[ref18] Gu Y. Zhang X. Tu S. Lindström K. (2009). Soil microbial biomass, crop yields, and bacterial community structure as affected by long-term fertilizer treatments under wheat-rice cropping. Eur. J. Soil Biol. 45, 239–246. doi: 10.1016/j.ejsobi.2009.02.005

[ref19] Hatfield J. L. Walthall C. L. (2015). Soil biological fertility: foundation for the next revolution in agriculture? Commun. Soil Sci. Plant Anal. 46, 753–762. doi: 10.1080/00103624.2015.1005227

[ref20] Ho A. Di Lonardo D. P. Bodelier P. L. E. (2017). Revisiting life strategy concepts in environmental microbial ecology. FEMS Microbiol. Ecol. 93:fix006. doi: 10.1093/femsec/fix006 28115400

[ref21] Hou S. Wolinska K. W. Hacquard S. (2021). Microbiota-root-shoot-environment axis and stress tolerance in plants. Curr. Opin. Plant Biol. 62:102028. doi: 10.1016/j.pbi.2021.102028, PMID: 33713892

[ref22] Hyakumachi M. (1994). Plant-growth-promoting fungi from turfgrass rhizosphere with potential for disease suppression. Soil Microorganisms 44, 53–68.

[ref23] Jangid K. Williams M. A. Franzluebbers A. J. Sanderlin J. S. Reeves J. H. Jenkins M. B.. (2008). Relative impacts of land-use, management intensity and fertilization upon soil microbial community structure in agricultural systems. Soil Biol. Biochem. 40, 2843–2853. doi: 10.1016/j.soilbio.2008.07.030

[ref24] Kennedy A. C. Smith K. L. (1995). Soil microbial diversity and the sustainability of agricultural soils. Plant Soil 170, 75–86. doi: 10.1007/BF02183056

[ref25] Leff J. W. Jones S. E. Prober S. M. Barberán A. Borer E. T. Firn J. L.. (2015). Consistent responses of soil microbial communities to elevated nutrient inputs in grasslands across the globe. Proc. Natl. Acad. Sci. U. S. A. 112, 10967–10972. doi: 10.1073/pnas.1508382112, PMID: 26283343 PMC4568213

[ref26] Liu Y. Zhou T. David C. Li L. Liu D. Zheng J.. (2012). Decline in topsoil microbial quotient, fungal abundance and C utilization efficiency of rice paddies under heavy metal pollution across South China. PLoS One 7:e38858. doi: 10.1371/journal.pone.0038858, PMID: 22701725 PMC3372496

[ref27] Lu H. Yan X. Zhu B. Zhang L. Feng X. Piao M.. (2020). The occurrence of peri-implant mucositis associated with the shift of submucosal microbiome in patients with a history of periodontitis during the first two years. J. Clin. Periodontol. 48, 441–454. doi: 10.1111/jcpe.13410 33617025

[ref28] Masunaka A. Hyakumachi M. Takenaka S. (2011). Plant growth-promoting fungus, *Trichoderma koningi* suppresses isoflavonoid phytoalexin vestitol production for colonization on/in the roots of *Lotus japonicus*. Microbes Environ. 26, 128–134. doi: 10.1264/jsme2.ME10176, PMID: 21502738

[ref29] Morris S. J. Blackwood C. B. (2015). “Soil microbiology, ecology and biochemistry,” in The Ecology of the Soil Biota and their Function. 4th Edn. (Salt Lake City, UT: Academic Press), 273–309.

[ref30] Nannipieri P. Ascher J. Ceccherini M. T. (2003). Microbial diversity and soil functions. Eur. J. Soil Sci. 54, 655–670. doi: 10.1046/j.1351-0754.2003.0556.x

[ref31] Newton R. J. Mcmahon K. D. (2011). Seasonal differences in bacterial community composition following nutrient additions in a eutrophic lake. Environ. Microbiol. 13, 887–899. doi: 10.1111/j.1462-2920.2010.02387.x, PMID: 21138514

[ref32] Pan G. Zhou P. Li Z. Smith P. Li L. Qiu D.. (2009). Combined inorganic/organic fertilization enhances N efficiency and increases rice productivity through organic carbon accumulation in a rice paddy from the tai Lake region. China. Agric. Ecosyst. Environ. 131, 274–280. doi: 10.1016/j.agee.2009.01.020

[ref33] Parham J. A. Deng S. P. Da H. N. Sun H. Y. Raun W. R. (2003). Long-term cattle manure application in soil. II. Effect on soil microbial populations and community structure. Biol. Fertil. Soils 38, 209–215. doi: 10.1007/s00374-003-0657-7

[ref34] Pianka E. R. (1970). On r- and K-selection. Am. Nat. 104, 592–597. doi: 10.1086/282697

[ref35] Qiu L. Zhang Q. Zhu H. Reich P. B. Banerjee S. van der Heijden M. G. A.. (2021). Erosion reduces soil microbial diversity, network complexity and multifunctionality. ISME J. 15, 2474–2489. doi: 10.1038/s41396-021-00913-1, PMID: 33712698 PMC8319411

[ref36] Quast C. Pruesse E. Yilmaz P. Gerken J. Schweer T. Yarza P.. (2013). The SILVA ribosomal RNA gene database project: improved data processing and web-based tools. Nucleic Acids Res. 41, D590–D596. doi: 10.1093/nar/gks1219, PMID: 23193283 PMC3531112

[ref37] Ramirez K. S. Lauber C. L. Knight R. Bradford M. A. Fierer N. (2010). Consistent effects of nitrogen fertilization on soil bacterial communities in contrasting systems. Ecology 91, 3463–3470. doi: 10.1890/10-0426.1, PMID: 21302816

[ref38] Rognes T. Flouri T. Nichols B. Quince C. Mahe F. (2016). VSEARCH: a versatile open source tool for metagenomics. Peer J. 4:e2584. doi: 10.7717/peerj.2584, PMID: 27781170 PMC5075697

[ref39] Saleem M. Hu J. Jousset A. (2019). More than the sum of its parts: microbiome biodiversity as a driver of plant growth and soil health. Annu. Rev. Ecol. Evol. Syst. 50, 145–168. doi: 10.1146/annurev-ecolsys-110617-062605

[ref40] Smit E. Leeflang P. Gommans S. van den Broek J. van Mil S. Wernars K. (2001). Diversity and seasonal fluctuations of the dominant members of the bacterial soil community in a wheat field as determined by cultivation and molecular methods. Appl. Environ. Microbiol. 67, 2284–2291. doi: 10.1128/AEM.67.5.2284-2291.2001, PMID: 11319113 PMC92868

[ref41] Sun H. Y. Deng S. P. Raun W. R. (2004). Bacterial community structure and diversity in a century-old manure-treated agroecosystem. Appl. Environ. Microbiol. 70, 5868–5874. doi: 10.1128/AEM.70.10.5868-5874.2004, PMID: 15466526 PMC522114

[ref42] Torsvik V. Øvreås L. (2002). Microbial diversity and function in soil: from genes to ecosystems. Curr. Opin. Microbiol. 5, 240–245. doi: 10.1016/S1369-5274(02)00324-7 12057676

[ref43] van der Bom F. Nunes I. Raymond N. S. Hansen V. Bonnichsen L. Magid J.. (2018). Long-term fertilisation form, level and duration affect the diversity, structure and functioning of soil microbial communities in the field. Soil Biol. Biochem. 122, 91–103. doi: 10.1016/j.soilbio.2018.04.003

[ref44] Wang J. Xue C. Song Y. Wang L. Huang Q. Shen Q. (2016a). Wheat and rice growth stages and fertilization regimes alter soil bacterial community structure, but not diversity. Front. Microbiol. 7:1207. doi: 10.3389/fmicb.2016.01207 27536292 PMC4971054

[ref45] Wang J. Zhang D. Zhang L. Li J. Raza W. Huang Q.. (2016b). Temporal variation of diazotrophic community abundance and structure in surface and subsoil under four fertilization regimes during a wheat growing season. Agric. Ecosyst. Environ. 216, 116–124. doi: 10.1016/j.agee.2015.09.039

[ref46] Wang J. Zheng J. Shi W. Du N. Xu X. Zhang Y.. (2018). Dysbiosis of maternal and neonatal microbiota associated with gestational diabetes mellitus. Gut 67, 1614–1625. doi: 10.1136/gutjnl-2018-315988, PMID: 29760169 PMC6109274

[ref47] Wu M. Qin H. Chen Z. Wu J. Wei W. (2011). Effect of long-term fertilization on bacterial composition in rice paddy soil. Biol. Fertil. Soils 47, 397–405. doi: 10.1007/s00374-010-0535-z

[ref48] Xie K. Z. Pei-Zhi X. U. Jiang R. P. Yu-Sheng L. U. Wen-Jie G. U. Wen-Ying L. I.. (2016). Combined application of inorganic and organic fertilizers improve rice yield and the abundance of soil nitrogen-cycling microbes in cold waterlogged paddy fields. J. Plant Nutr. Fertilizer 22, 1267–1277. doi: 10.11674/zwyf.15306

[ref49] Xun W. Zhao J. Xue C. Zhang G. Ran W. Wang B.. (2016). Significant alteration of soil bacterial communities and organic carbon decomposition by different long-term fertilization management conditions of extremely low-productivity arable soil in South China. Environ. Microbiol. 18, 1907–1917. doi: 10.1111/1462-2920.13098, PMID: 26486414

[ref50] Yu L. Nicolaisen M. Larsen J. Ravnskov S. (2013). Organic fertilization alters the community composition of root associated fungi in *Pisum sativum*. Soil Biol. Biochem. 58, 36–41. doi: 10.1016/j.soilbio.2012.11.004

[ref51] Yuan G. Zhang J. B. Zhang L. M. Min Y. He J. Z. (2008). Long-term fertilization regimes affect bacterial community structure and diversity of an agricultural soil in northern China. J. Soils Sediments 8, 43–50. doi: 10.1065/jss2008.01.270

[ref52] Zhang J. Liu Y. X. Zhang N. Hu B. Jin T. Xu H.. (2019). NRT1.1B is associated with root microbiota composition and nitrogen use in field-grown rice. Nat. Biotechnol. 37, 676–684. doi: 10.1038/s41587-019-0104-4, PMID: 31036930

[ref53] Zhang J. Zhang N. Liu Y. X. Zhang X. Hu B. Qin Y.. (2018). Root microbiota shift in rice correlates with resident time in the field and developmental stage. Sci. China Life Sci. 61, 613–621. doi: 10.1007/s11427-018-9284-4, PMID: 29582350

[ref54] Zhao J. Ni T. Li J. Lu Q. Fang Z. Huang Q.. (2016). Effects of organic–inorganic compound fertilizer with reduced chemical fertilizer application on crop yields, soil biological activity and bacterial community structure in a rice–wheat cropping system. Appl. Soil Ecol. 99, 1–12. doi: 10.1016/j.apsoil.2015.11.006

[ref55] Zhao J. Ni T. Li Y. Xiong W. Ran W. Shen B.. (2014). Responses of bacterial communities in arable soils in a rice-wheat cropping system to different fertilizer regimes and sampling times. PLoS One 9:e85301. doi: 10.1371/journal.pone.0085301, PMID: 24465530 PMC3896389

[ref56] Zhou J. Guan D. Zhou B. Zhao B. Ma M. Qin J.. (2015). Influence of 34-years of fertilization on bacterial communities in an intensively cultivated black soil in Northeast China. Soil Biol. Biochem. 90, 42–51. doi: 10.1016/j.soilbio.2015.07.005

