# Supplementary material for: Long-Term Organic–Inorganic Fertilization Regimes Alter Bacterial and Fungal Communities and Rice Yields in Paddy Soil
Source: Front Microbiol. 2022 Jun 27;13:890712. doi: 10.3389/fmicb.2022.890712 (PMC9271892; doi:10.3389/fmicb.2022.890712)
Supplement: Supplementary file 1 [file Image_1.pdf]

**Supplement Figure**

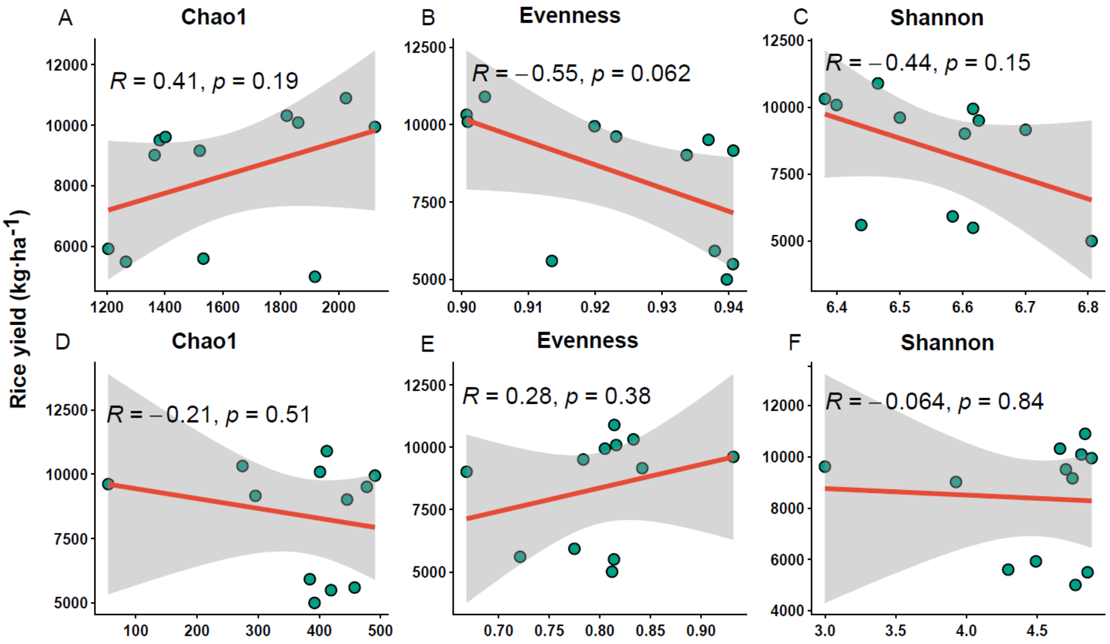


**Figure S1.** Correlation analyses between indices of bacterial and fungal alpha diversity and rice yield. **(A-C)** Correlations between bacterial indices of α diversity and rice yield. **(D-F)** Correlations between of fungal indices of α diversity and rice yield.
